# Wild-Type Yellow Fever Virus RNA in Cerebrospinal Fluid of Child

**DOI:** 10.3201/eid2508.181479

**Published:** 2019-08

**Authors:** Paula E.S. Marinho, Pedro P.M. Alvarenga, Ana P.C. Crispim, Talitah M.S. Candiani, Alice M. Alvarenga, Isabela M. Bechler, Pedro A. Alves, Fabio P. Dornas, Danilo B. de Oliveira, Aline A. Bentes, Paulo P. Christo, Erna G. Kroon

**Affiliations:** Universidade Federal de Minas Gerais, Belo Horizonte, Brazil (P.E.S. Marinho, A.P.C. Crispim, A.A. Bentes, E.G. Kroon);; Hospital Infantil João Paulo II, Belo Horizonte (P.P.M. Alvarenga, T.M.S. Candiani, A.M. Alvarenga, I.M. Bechler, A.A. Bentes);; Instituto René Rachou, Belo Horizonte (P.A. Alves);; Universidade Federal dos Vales do Jequitinhonha e Mucuri, Diamantina, Brazil (F.P. Dornas, D.B. de Oliveira);; Instituto de Ensino e Pesquisa Santa Casa de Belo Horizonte, Belo Horizonte (P.P. Christo)

**Keywords:** Yellow fever virus, yellow fever virus RNA, viruses, flavivirus, wild-type virus, yellow fever, child, vector-borne infections, mosquito-borne infections, cerebrospinal fluid, CSF, central nervous system, CNS, meningitis/encephalitis

## Abstract

We report a 3-year-old child who was hospitalized because of severe manifestations of the central nervous system. The child died after 6 days of hospitalization. Analysis of postmortem cerebrospinal fluid showed the presence of yellow fever virus RNA. Nucleotide sequencing confirmed that the virus was wild-type yellow fever virus.

Yellow fever virus (family *Flaviviridae*, genus *Flavivirus*) is spread by bites of infected mosquitoes of the genera *Aedes*, *Haemagogus*, and *Sabethes* (*1*). During 2017, an epidemic of yellow fever in Brazil resulted in 1,266 confirmed human cases and 415 deaths; 532 confirmed cases and 181 deaths occurred in Minas Gerais State (*2*). We report wild-type yellow fever virus RNA in the cerebrospinal fluid of a child who died during this outbreak.

## The Study

In May 2017, a 3-year-old girl born on February 13, 2014, and who lived in Belo Horizonte, Minas Gerais, Brazil, was hospitalized because of influenza-like signs and symptoms that started 2 days earlier. Signs and symptoms included nasal congestion and discharge, sneezing, and fever. The medical history included no seizures or epilepsy, family history of neurologic diseases, or allergies. Immunizations for the patient were current and included 1 dose of yellow fever vaccine (vaccine lot 136VFA022Z, expiration date June 30, 2015), given at 10 months of age on December 18, 2014, following the vaccination schedule of the Ministry of Health of Brazil.

During hospitalization, the patient showed a loss of consciousness and several episodes of seizures. She was given intravenous midazolam and phenobarbital for status epilepticus. During one of the seizures, the patient had cardiac arrest; she was given cardiac resuscitation for 12 min and transferred to an intensive care unit because of hemodynamic instability. Only brain stem reflexes were detected. Ceftriaxone and acyclovir were given as empiric treatment for possible meningoencephalitis. No lumbar puncture or computed tomography of the brain were performed because the patient was clinically unstable. 

On day 1 after admission, we performed liver biochemical function tests. These tests showed the following levels: alanine aminotransferase 67 U/L, aspartate aminotransferase 109 U/L, direct bilirubin 0.49 mg/dL, indirect bilirubin 0.34 mg/dL, alkaline phosphatase 125 U/L, γ-glutamyl transpeptidase 26 U/L, and albumin 2.1 mg/dL.

On day 2 of hospitalization, the patient still had a severe hemodynamically unstable condition that required constant adjustments in amine doses (noraderenalin 2 μg/kg/min and dobutamin 8 μg/kg/min), but she showed spontaneous opening of the eyes and nonspecific thoracic movements. No major metabolic or hydroelectrolytic disturbances were detected in the time since her hospitalization. No signs of hemorrhage and hemostatic disorders were detected.

Despite the therapeutic measures, on the third day of hospitalization, the child showed a severe worsening of her condition, which included fixed mydriasis, absence of corneopalpebral and oculocephalic reflexes, and no reflex of coughing during aspiration of the orotracheal tube. The Glasgow Coma Scale score was 3. The child had deep hypotonia and areflexia and no clonus during her examination. Funduscopic examination showed bilateral papilledema. Congruent with these findings, computed tomography of the brain showed diffuse cerebral edema with erasure of basement cisterns and imminent signs of herniation.

**Figure 1:**
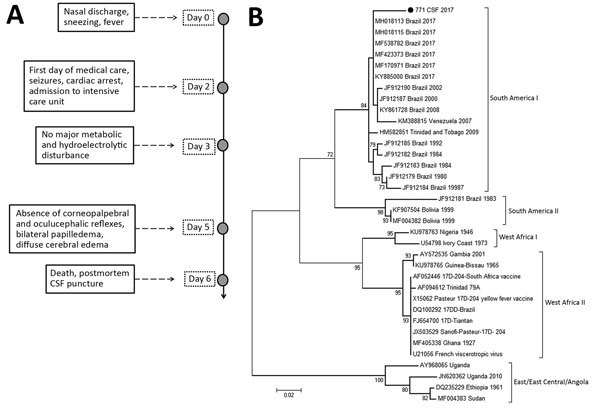
Illness timeline and phylogenetic testing in case of child with wild-type yellow fever virus RNA in CSF, Brazil, 2017. A) Timeline of symptoms, ambulatory procedures, and laboratory results. B) Phylogenetic tree of yellow fever viruses. The tree was constructed by using the maximum-likelihood method and the Tamura-Nei model in MEGA 7.0 software (https://www.megasoftware.net). Numbers to the left of nodes are bootstrap values (1,000 replicates). Sequences were compared with sequences in GenBank; black dot indicates sequence isolated in this study. This sequence was deposited in GenBank (accession no. 771_2017_CSF_NS5 MK450540). Scale bar indicates nucleotide substitutions per site. CSF, cerebrospinal fluid.

Even after institution of neuroprotection measures (3% saline solution and mannitol), the patient showed a worsening of her condition, which included multiple organ dysfunction, refractory shock to the use of vasoactive drugs, diabetes insipidus, and deterioration of glycemic control. The patient died 6 days after her hospitalization. After obtaining permission of her parents, we collected a postmortem CSF sample immediately after her death (Figure 1, panel A).

Postmortem CSF biochemical analyses showed glucose 117 mg/dL, protein 434 mg/dL, lactate 6.8 mmol/L, and leukocyte count 18 cells/mm^3^ (61% lymphocytes, 4% neutrophils, and 24% monocytes). We tested the CSF by using a quantitative PCR for DNA and RNA viruses commonly associated with central nervous system (CNS) infections, such as herpesvirus, enteroviruses, flaviviruses (dengue virus, Zika virus, St. Louis encephalitis virus, West Nile virus, and yellow fever virus), and chikungunya virus. The CSF showed negative results for other potential neurotropic viruses and meningococcus, *Haemophilus* spp., and pneumococcus when tested by a reference laboratory in Minas Gerais. However, the CSF was positive for yellow fever virus RNA according to the protocol reported by Domingo et al. (*3*). Results were negative for the other virus tested by a quantitative PCR. We performed a quantitative PCR specific for flavivirus nonstructural protein 5 gene (*4*) and obtained a DNA fragment that we used directly for nucleotide sequencing.

**Figure 2:**
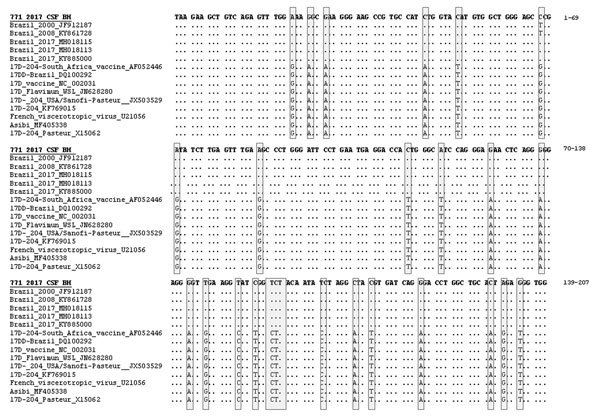
Alignment of a fragment of yellow fever virus 771_2017_CSF_ NS5 sequence (207 bp) from child with wild-type yellow fever virus in CSF, Brazil, 2017, with other yellow fever virus sequences. Sequences were obtained from GenBank and aligned by using standard parameters of ClustalW (http://www.clustal.org). Shaded boxes indicate major variations among wild-type virus sequences and vaccine virus sequences. Dots indicate sequence identity. BH, Belo Horizonte; CSF, cerebrospinal fluid.

We aligned the sequence and used it to construct a phylogenetic tree by using the maximum-likelihood method (Figure 1, panel B). Phylogenetic analysis showed that sequence grouped with other wild-type yellow fever virus sequences from Brazil in a clade separate from vaccine virus samples, and the alignment confirmed a sequence difference of 25 nt (Figure 2). We performed a plaque reduction neutralization test with CSF and obtained a negative result.

## Conclusions

We detected wild-type yellow fever virus RNA by reverse transcription PCR in the CSF of a previously healthy child who died during a yellow fever outbreak in Brazil. Virus RNA was detected 2 days after manifestation of mild influenza-like signs and symptoms, sensory impairment, and recurrent epileptic seizures. We detected no hemorrhagic, metabolic, or hydroelectrolytic changes in this patient, and test results for other pathogens, such as bacteria and neurotropic viruses, were negative.

Neurologic involvement in wild-type yellow fever virus infection was described in a study conducted in Nigeria during a 1969 epidemic in which 103 patients were given a diagnosis of yellow fever, including 14 children and adolescents <19 years of age (*5*). In that study, signs and symptoms of CNS involvement were reported in 26 (25%) patients, 8 of whom had generalized seizures. CNS involvement was also reported in Nigeria during 1990 (*6*), and altered mental state was described in >61% of the patients in a yellow fever outbreak in Uganda during 2016 (*7*).

In a study in Darfur, Sudan, during a 2012 yellow fever epidemic, 844 cases of suspected yellow fever were reported (*8*). Children <15 years of age accounted for 21.4% of these case-patients, and 15.6% of them had seizures. In the same study, ≈8% of case-patients were reported to have been vaccinated, suggesting that the immunity caused by the vaccine might be affected by other factors that influence the effectiveness and duration of the vaccine (*8*).

Meningoencephalitis caused by yellow fever virus vaccine–associated neurotropic disease has been frequently reported in infants and adults (*1*,*9*,*10*). However, in our case report, the child had received 1 dose of 17D yellow fever vaccine at 10 months of age, which was 29 months before onset of signs and symptoms of encephalitis. The reported seroconversion rate of the 17D vaccine in this age group is only 72% (*11*). It is possible that this child was within the small percentage of vaccine failures because she had no history of previous hospitalizations or concurrent conditions that indicated immunosuppression.

Our patient had no classical signs or symptoms of yellow fever. Neurologic manifestations after infections with other flaviviruses are also often not accompanied by classic signs or symptoms of infection (*12*,*13*). Because signs and symptoms of encephalitis preceded cardiac arrest in our patient, it is unlikely that the virus infection occurred because of hemodynamic instability and cardiac arrest, which might increase the permeability of the blood–brain barrier and enable passage of the virus into the CNS. Despite scarce description of neurologic diseases in humans caused by wild-type yellow fever virus, a study with animal models confirmed that yellow fever virus is neurotropic and leads to fatal encephalitis (*14*).

The nucleotide sequence of the virus from the patient in our study was also identical to that of the virus that circulated during an outbreak of yellow fever in Belo Horizonte, Brazil (*15*). Although yellow fever encephalitis is rare, it was the primary neurologic manifestation in our patient.
